# Medical News and Bulletin

**Published:** 1843-12-09

**Authors:** 


### MEDICAL NEWS AND BULLETIN.

### JEFFERSON MEDICAL COLLEGE.

We understand that there are over three hundred matriculants at this Institution, the present season.

### THE CHARACTERISTICS OF THE ITALIAN SCHOOL.

In Italy, at present, there are very few men, who apply themselves with diligence to the patient investigation of details upon any particular subject of inquiry. Most of the physicians have adopted certain general principles of medical doctrine; and on these they base their practice. Hence it is that almost all their treatises assume the synthetic form, and that, even in their works of elementary instruction, the exposition and description of details are too often sacrificed to the abstract generalities of an ingenious dogmatism.

In some districts of Italy, and especially at Naples and Modena, Hippocratism is altogether prevalent. In the latter city, indeed, the Faculty of Medicine may be considered as an association less for instruction in modern medicine than for perpetuating the doctrines of the Coan sage. The orthodoxy of the school is based upon them; everything is redolent of antiquity; the students are taught to regard the Aphorisms as the oracles of medical truth; when they aspire to the doctorate, they are called upon to expound and illustrate one or more of these pithy sayings; and it is with their hands, laid upon the works of the old Greek, that they take the oath upon the day of their inauguration.

While the doctrines of the Hippocratic Medicine, more or less liberally interpreted, are almost universally received through the whole of the south of Italy, the northern half of the Peninsula has long been agitated by the disputed tenets of the Controstimulism. Turin, Pavia, and especially Milan, are the head-quarters of this school. *Rasori* its founder started, as is well known, as a disciple of the Brunonian doctrines, and was the translator of the Scotch physician's work. All the phenomena of disease he strove to reduce to one simple morbid action, viz. excessive or deficient stimulation; and like every other advocate of an exclusive system of pathology, he fell into the most egregious blunders, which proved to be injurious alike to sound theory and to safe practice. All diseases he considered to be systemic or general; and local inflammations therefore be looked upon as mere complications or accidental phenomena—the very reverse of the Broussaian creed. Professor *Tommasini* of Parma, adopted a sort of intermediate doctrine between these conflicting systems. Assenting to the general principle of his fellow-countryman, *Rasori*, he was too sagacious not to observe that local irritation, when extensive and long continued, is apt to give rise to constitutional diseases. Let us not deprive this distinguished physician of his due; it is right to acknowledge that he clearly enunciated the importance of local affections, as the cause of many diseases, before *Broussais* had published a line upon the subject.—*Combes on Italian Medicine, from Medico-Chirurgical Review*, *Oct.* 1843.

### PROFESSIONAL SUCCESS—GRAVITY VERSUS HILARITY.

Our contemporary, the Gazette, has favoured the profession with an Essay on Professional Success, which is good in its way, though not exactly after the manner of Dean Swift, nor Rabelais, nor Goldsmith, nor even quite so light as Addison's. But *n'importe*, it inculcates some very souud decorum and morality, and, if somewhat prosy, shares that fault in common with many excellent discourses. But the point to which we intend to allude is, one generally discussed by our contemporary—the advantages of seriousness. He observes:

"Seriousness, not occasional but habitual; earnestness, not affected but real, are useful in securing the confidence of the sick, and indispensable in the studies which prepare us for practice: and if the unvarying liveliness of a mercurial temperament, seems, in some instances, to have been a prime element of professional success, we may rely on it that such unwearied cheerfulness has only lightened the labours of conscientious activity; that it has been kept up by a consciousness of duties performed, not used as a substitute for duties neglected. Of those who can set the table in a roar, how many can leave it at the calls of duty?"

True as this may be, it cannot be deemed new, as their very charter designates the members of the College of Physicians "viri *tristes* et docti," and, no doubt, their solemnity stands them in stead. Who has not remarked some learned man, bending over a dubious stool, with a face in which are displayed all that seriousness and earnestness, so powerfully inculcated by our contemporary; and who, at the same time, has not noted the admiration which such philosophical devotedness to the cause of science and his patient, has excited in the attendant mother, or the aunt, or, above all, in the grandmother? The well weighed words, the Burleigh shake, of a professional opinion, as dark as the darkest Delphic oracle, and quite as well suited to whatever may turn up, are parts of that gravity which must pervade the sayings and doings of a doctor, and to the uninitiated savour a little of humbug. In fact it is often, in this composite world of ours, difficult to separate the latter abused, but well used, instrument from the "conscientious earnestness" which it represents.

It must be agreed, we think, *nem. con.*, that doctors of physic should be grave, at any rate before their patients. Who, then, is to be lively? for something like fun may surely be permitted to the faculty. We fancy it must be given up to those queer fellows, the surgeons—whose business of passing catheters, tying piles, and curing claps, does not necessarily require such length of visage, or intense profundity of thought. And hence, we suppose it is, that so many of these same surgeons have been chatty, good-humoured sorts of men, not indisposed to crack either a bottle, or a joke, and have not greatly suffered in the estimation of the world notwithstanding. Far be it from us to defend this. We merely chronicle the fact, and at the present time of day, when to look wise is almost as good as to be so, and to scorn pleasantry is held by many as a certain evidence of mind, we would caution the rising generation against being misled by the lives of such persons as Cooper or Abernethy.—*Medico-Chirurgical Review*, Oct. 1843.

